# Towards High-Efficiency Inverted CH_3_NH_3_GeI_3_ Perovskite Solar Cells

**DOI:** 10.3390/ma19132700

**Published:** 2026-06-23

**Authors:** Hong-Tao Li, Kang Yan, Jin Wang, Shuang-Shuang Zhang, Peng-An Zong, Xiao-Dong Feng

**Affiliations:** College of Materials Science and Engineering, Nanjing Tech University, Nanjing 211816, China; 202461103115@njtech.edu.cn (H.-T.L.); lht0414nltdz@gmail.com (K.Y.); 202361103043@njtech.edu.cn (J.W.); zhangshuangshuang@njtech.edu.cn (S.-S.Z.); pazong@njtech.edu.cn (P.-A.Z.)

**Keywords:** perovskite solar cell, MAGeI_3_, SCAPS, simulation

## Abstract

The performance of inverted CH_3_NH_3_GeI_3_ (MAGeI_3_) perovskite solar cells incorporating both a hole transport layer (HTL) and an electron transport layer (ETL) was investigated using the Solar Cell Capacitance Simulator (SCAPS). Three candidate HTLs, including PEDOT:PSS, MoS_2_, and WS_2_, along with five ETLs including PCBM, TiO_2_, IGZO, ZnO, and SnO_2_, have been systematically evaluated. The analysis shows that WS_2_ and SnO_2_ provided the most favorable hole and electron transport, respectively. To improve device efficiency, the absorber layer thickness, defect density in MAGeI_3_, doping levels of WS_2_ and SnO_2_, as well as the interface defect densities and the work function of indium tin oxide (ITO), have been systematically studied. The optimal absorber layer thickness is determined to be approximately 900 nm. The optimal doping density of both WS_2_ and SnO_2_ is 1 × 10^19^ cm^−3^. The MAGeI_3_ layer should maintain a defect density as low as 1 × 10^15^ cm^−3^, and the defect densities at MAGeI_3_ interfaces should remain at 1 × 10^15^ cm^−2^. Additionally, an ITO work function of at least 5.2 eV is necessary to prevent the formation of a Schottky barrier at the ITO/WS_2_ interface. The simulated power conversion efficiency (PCE) can reach 22.9% under these optimized conditions. Our simulation results offer a viable route to develop high-efficiency MAGeI_3_ perovskite solar cells.

## 1. Introduction

Organic–inorganic hybrid perovskites have attracted considerable attention as photovoltaic absorbers because of their excellent optoelectronic properties, such as strong light absorption, efficient carrier transport, long carrier diffusion length, and adjustable band gaps [1,2,3,4]. Since perovskite solar cells (PSCs) were first reported in 2009 [5], continuous advances in material design, film preparation, and device engineering have led to a rapid improvement in their photovoltaic performance, particularly the power conversion efficiency (PCE) [6,7,8,9,10]. During the past 17 years, the PCE of PSCs has improved remarkably, increasing from the initial efficiency of 3.8% to a certified record value of 27.3% [5,11]. Currently, lead-based perovskite materials with an ABX_3_ crystal structure have been broadly utilized as the light-absorbing layer in high-efficiency PSCs. As monovalent cations, CH_3_NH_3_^+^(MA^+^) and HC(NH_2_)^2+^(FA^+^) can occupy position A. Position B can be occupied by divalent metal ions, with Pb^2+^ being a typical example; position X is occupied by halide anions, such as Cl^−^, Br^−^, and I^−^ [12,13]. Although the PCE of Pb-based PSCs has been improved substantially, one serious problem of PSCs is that the toxicity of Pb can handicap their potential application by causing serious harm to environmental safety and human well-being [14]. Consequently, developing Pb-free PSCs is essential to achieve photovoltaic performance comparable to that of Pb-based PSCs [15,16]. As elements in the same group as Pb in the periodic table, germanium (Ge) and tin (Sn) are considered viable in the perovskite structure [17,18]. Sn-based perovskites have received extensive attention and have achieved much higher efficiencies than Ge-based perovskites [19,20,21]. However, Sn^2+^ can be oxidized to Sn^4+^ during film formation and device operation, which is usually accompanied by tin vacancies, high background carrier concentration, and increased nonradiative recombination [22]. Ge-based perovskites, such as CH_3_NH_3_GeI_3_ (MAGeI_3_), are also Pb-free and may reduce the toxicity concern associated with Pb-based PSCs [17,23]. Compared with Sn-based perovskites, MAGeI_3_ is still at a much earlier stage of development. In 2015, Krishnamoorthy et al. first fabricated MAGeI_3_-based solar cells, and their PCE was only 0.20% [23]. The main challenges of MAGeI_3_-based PSCs include limited experimental efficiency, difficulty in preparing high-quality films, and possible oxidation of Ge-containing perovskites under ambient conditions [17,23]. Therefore, although Ge oxidation has received less attention than the well-known Sn^2+^/Sn^4+^ oxidation in Sn-based perovskites, it should still be regarded as an important stability issue for MAGeI_3_-based devices. Since only limited experimental progress has been reported for MAGeI_3_ solar cells, numerical simulation is useful for identifying key parameters and guiding future device optimization.

Planar heterojunctions have become the mainstream structure for perovskite solar cells. This structure is fundamentally divided into two types: the conventional structure (n-i-p) and the inverted structure (p-i-n). Compared to the conventional structure, the inverted structure can be fabricated at lower temperatures through a more simplified process, thereby achieving superior stability and reduced current hysteresis effects [24,25,26]. Inverted structures are widely employed in Pb-based perovskite solar cells. Jiang et al. [27] reported an inverted device achieving a maximum PCE of 25.5%.

Theoretically, researchers have studied the normal structure of MAGeI_3_-based PSCs with different hole transport layers (HTLs) and electron transport layers (ETLs) [28,29,30,31], as summarized in Table 1. However, theoretical investigations of inverted MAGeI_3_-based perovskite solar cells remain scarce [32].

The previous simulation studies listed in Table 1 mainly focused on normal MAGeI_3_-based device configurations or on the influence of selected transport layers. However, the performance of inverted MAGeI_3_ solar cells is not determined by a single layer parameter, but by the combined effects of carrier-selective contacts, interfacial band offsets, absorber quality, transport-layer doping, interface recombination, and electrode work function. Therefore, a more integrated analysis is still required to clarify how these factors jointly govern the device performance of inverted MAGeI_3_-based PSCs.

In this work, an inverted MAGeI_3_ perovskite solar cell structure is investigated using the Solar Cell Capacitance Simulator (SCAPS). Indium tin oxide (ITO) is used as the front electrode for hole collection, while silver (Ag) serves as the back electrode for electron collection [33]. PEDOT:PSS is considered because it has been widely used as an HTL in inverted PSCs [34]. MoS_2_ and WS_2_ are examined as inorganic HTLs because two-dimensional transition metal dichalcogenides (TMDs) have been investigated as functional semiconductor layers in perovskite-related optoelectronic devices [35]. In particular, thin MoS_2_ films and water-soluble transition metal dichalcogenides have been used as hole transport layers in planar or p-i-n PSCs [36,37]. WS_2_ is further considered because WS_2_ thin films show useful optoelectronic properties, including electrical conductivity, which is relevant to carrier transport in solar-cell devices [38]. In addition, WS_2_/perovskite heterostructures have been reported to influence perovskite film growth and interfacial charge behavior through van der Waals interactions [39,40,41]. For electron extraction, PCBM, TiO_2_, IGZO, ZnO, and SnO_2_ are compared as candidate ETLs, since the ETL plays an important role in electron collection and interfacial recombination control [42]. Different from previous studies that mainly optimized limited structural parameters, this work evaluates the effects of HTL/ETL selection, absorber thickness, transport-layer doping density, absorber defect density, interfacial defect density, and ITO work function within the same inverted device framework. This combined optimization provides a clearer understanding of the physical factors limiting MAGeI_3_-based inverted PSCs and identifies parameter ranges that may guide further experimental development.

## 2. SCAPS Simulation Methodology and Device Arrangements

### 2.1. Numerical Method

SCAPS (version 3.3.11), developed by Burgelman et al. at Ghent University [43], is a widely utilized tool for simulating solar cells. The software operates based on Poisson’s equation (Formula (1)), the continuity equations (Formula (2)), and the drift-diffusion equations (Formula (3)). By solving these equations under defined boundary conditions, SCAPS establishes a quantitative relationship between current density and voltage, enabling the determination of fundamental performance parameters of solar cells.

(1) $$\frac{{𝜕}^{2}\varphi }{𝜕x^{2}}=\frac{q}{\epsilon }\left(n-p\right)$$

(2) $$\frac{{𝜕}_{n}}{𝜕t}=\frac{1}{q}\frac{𝜕J_{n}}{{𝜕}_{x}}+G-R\;\frac{{𝜕}_{p}}{𝜕t}=-\frac{1}{q}\frac{𝜕J_{p}}{{𝜕}_{x}}+G-R$$

(3) $$J_{n}=qD_{n}\frac{𝜕n}{𝜕x}-q\mu_{n}n\frac{{𝜕}_{\varphi }}{{𝜕}_{x}}\;J_{p}=-qD_{p}\frac{𝜕p}{𝜕x}-q\mu_{p}p\frac{{𝜕}_{\varphi }}{{𝜕}_{x}}$$

### 2.2. Device Structure

**Figure 1:**
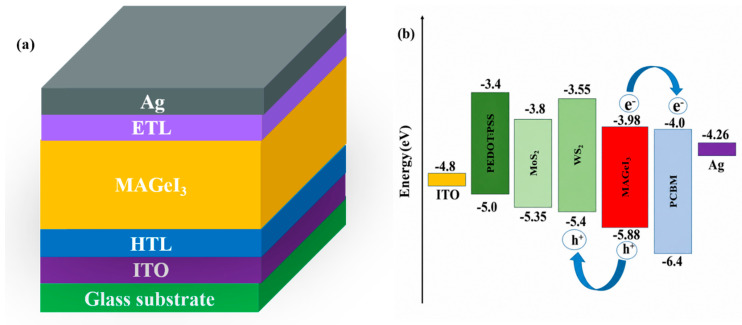
(**a**) Schematic illustration of the MAGeI_3_-based inverted PSCs structure, (**b**) band alignment diagram of PSCs.

The device structure illustrated in Figure 1a is glass substrate/ITO/HTL/MAGeI_3_/ETL/Ag, where the HTL includes MoS_2_, WS_2_, and PEDOT:PSS, and the ETL includes PCBM, IGZO, TiO_2_, ZnO, and SnO_2_. Initially, PCBM is selected as an ETL, which is widely used in PSCs, and the corresponding energy level alignment is presented in Figure 1b.

### 2.3. Material Parameters

The parameters adopted for the device simulations were collected from the reported literature and the SCAPS material database [44,45,46,47], as listed in Table 2. Table 3 presents the defect parameters at the MAGeI_3_ interfaces, while Table 4 lists the initial material parameters of the ETLs. The optical absorption of MAGeI_3_ was described using the default SCAPS absorption model, in which the absorption coefficient follows a square-root dependence on photon energy above the bandgap, with E_g_ = 1.90 eV. The default illumination conditions are AM1.5G and a 300 K temperature.

**Figure 2:**
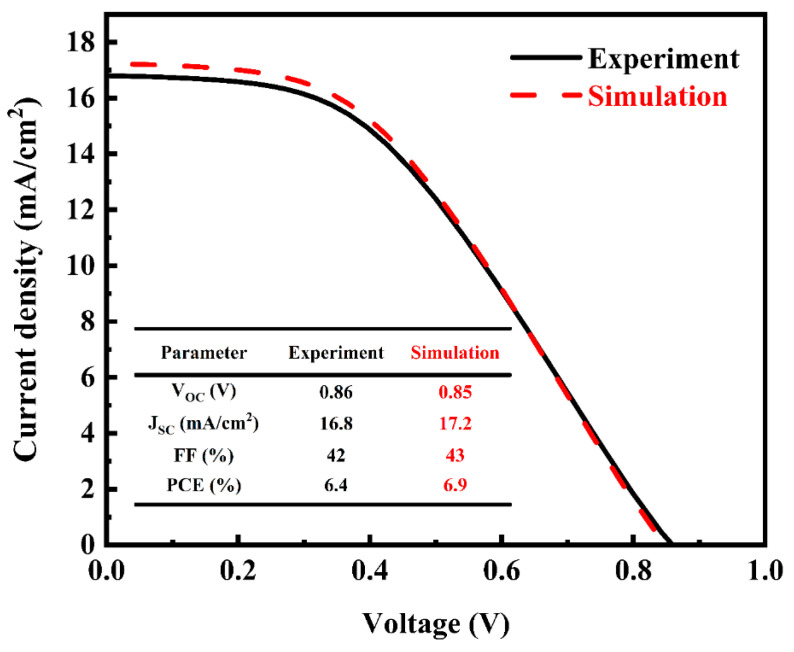
Comparison of experiment and simulation data of PSCs.

To validate the simulation parameters, we simulated a reported MASnI_3_ device made by Noel et al. [48]. Figure 2 shows that the simulated current–voltage (J-V) characteristics closely match the experimental data, indicating that the initial simulation parameters are reasonable.

## 3. Results and Discussion

### 3.1. Impact of Different HTLs

**Figure 3:**
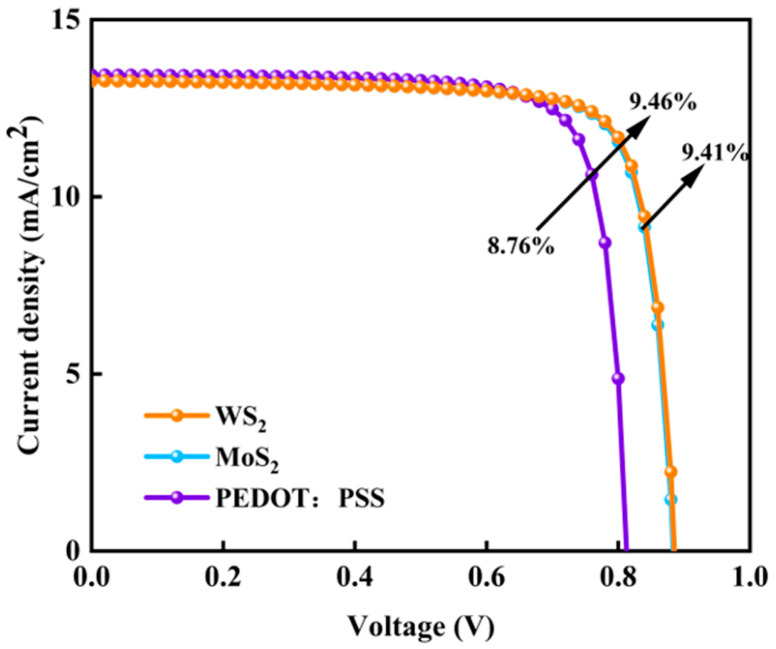
Effect of HTL selection on the J-V characteristics of PSCs.

Figure 3 compares the current density voltage (J-V) characteristics of devices using PCBM as the ETL and WS_2_, MoS_2_, or PEDOT:PSS as the HTL. Table 5 shows that the WS_2_-based device gives the highest simulated PCE of 9.46% among the three investigated HTLs. Its open-circuit voltage (V_OC_), short-circuit current density (J_SC_), and fill factor (FF) are 0.89 V, 13.28 mA/cm^2^, and 80.04%, respectively. These results indicate that the HTL strongly affects the photovoltaic performance through band alignment and carrier selectivity at the MAGeI_3_/HTL interface. Previous band offset studies have shown that proper valence-band offset (VBO) regulation at the perovskite/HTL interface is important for efficient hole extraction and recombination suppression [49,50]. Among the investigated HTLs, the WS_2_ device shows the highest simulated V_OC_ and PCE. The higher V_OC_ can be related to reduced interfacial recombination, while the comparable FF values indicate that carrier extraction remains effective at the MAGeI_3_/WS_2_ interface. The J_SC_ values of the three devices are relatively close, suggesting that the HTL mainly influences carrier collection rather than optical absorption under the same absorber thickness. As shown in Figure 1b, WS_2_ forms a favorable VBO with MAGeI_3_, which facilitates hole transport. In addition, the larger conduction band barrier between WS_2_ and MAGeI_3_ helps block electrons and suppress interfacial recombination. Therefore, WS_2_ is selected as the HTL for the following analysis under the present simulation conditions.

### 3.2. Impact of Different ETLs

**Figure 4:**
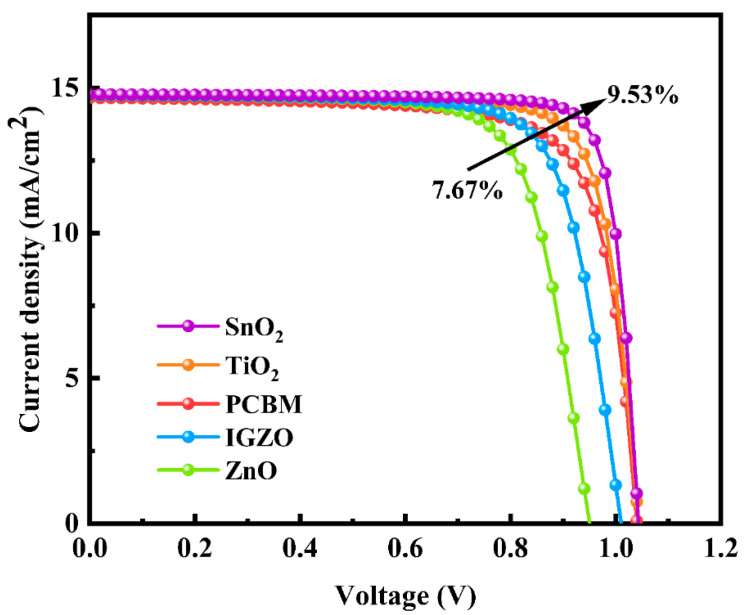
Effect of ETL selection on the J-V characteristics of PSCs.

**Figure 5:**
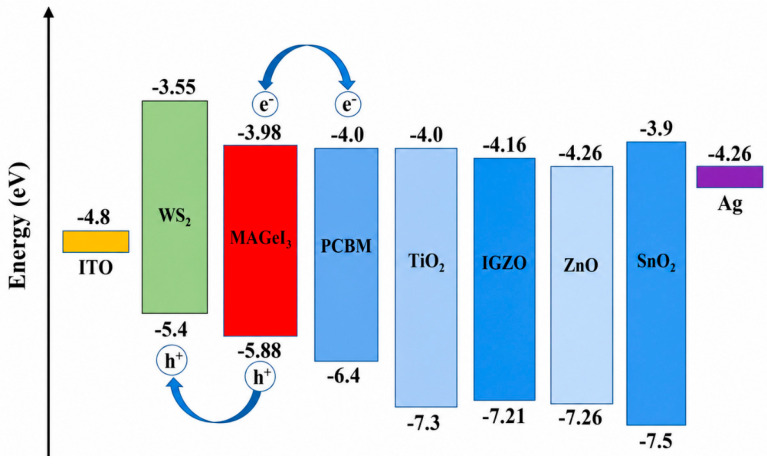
Energy level alignment of PSCs employing different ETLs.

The J-V curves in Figure 4 compare the devices employing different ETLs, including PCBM, TiO_2_, IGZO, ZnO, and SnO_2_, and their band alignment diagrams are shown in Figure 5. Table 6 shows that the SnO_2_-based device gives the highest simulated PCE of 9.53% among the five investigated ETLs. As shown in Table 6, the J_SC_ values of the devices with different ETLs remain close, indicating that the ETL has a limited influence on photocurrent generation under the same MAGeI_3_ absorber thickness. In contrast, V_OC_, FF, and PCE show more obvious variations, suggesting that the ETL mainly affects device performance by modulating electron extraction and interfacial recombination at the ETL/MAGeI_3_ interface.

**Figure 6:**
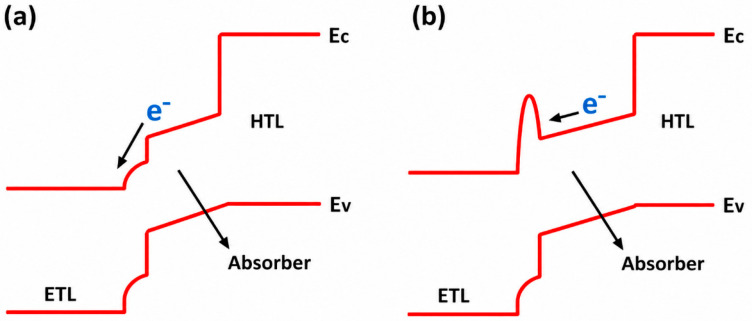
Schematic comparison of (**a**) cliff-type and (**b**) spike-type band offsets.

The conduction-band offset (CBO) across the ETL/MAGeI_3_ junction can influence carrier recombination [51,52]. CBO is determined by the conduction-band energy difference across the ETL/perovskite junction. Previous studies have shown that CBO optimization is important for selecting suitable transport layers in MAGeI_3_ solar cells and for improving J_SC_, V_OC_, FF, and PCE through band alignment control [50]. As shown in Figure 6a, a negative CBO results in a cliff-like band alignment when the conduction-band minimum of the ETL is lower than that of the perovskite absorber. In contrast, a positive CBO corresponds to a spike-like band alignment, as illustrated in Figure 6b. Typically, a cliff structure can accumulate more electrons at the interface, accelerating carrier recombination, leading to a decrease in V_OC_ and PCE [53]. The ETL of ZnO shows the lowest efficiency because the largest cliff-like structure (−0.28 eV) is formed at the interface. When the barrier of a low spike structure (e.g., CBO = 0.1 eV) is formed, the interfacial carrier recombination can be diminished, and the device performance will be improved [28]. At the SnO_2_/MAGeI_3_ interface, a small spike-like barrier of 0.08 eV is established, which helps suppress interfacial recombination and contributes to the improved PCE. Moreover, SnO_2_ exhibits a relatively large VBO with respect to MAGeI_3_ and thus acts as a hole reflector, which suppresses recombination and improves device performance. Therefore, SnO_2_ is selected as the ETL for the subsequent simulations under the present simulation conditions.

### 3.3. Performance Dependence on Absorber Thickness

**Figure 7:**
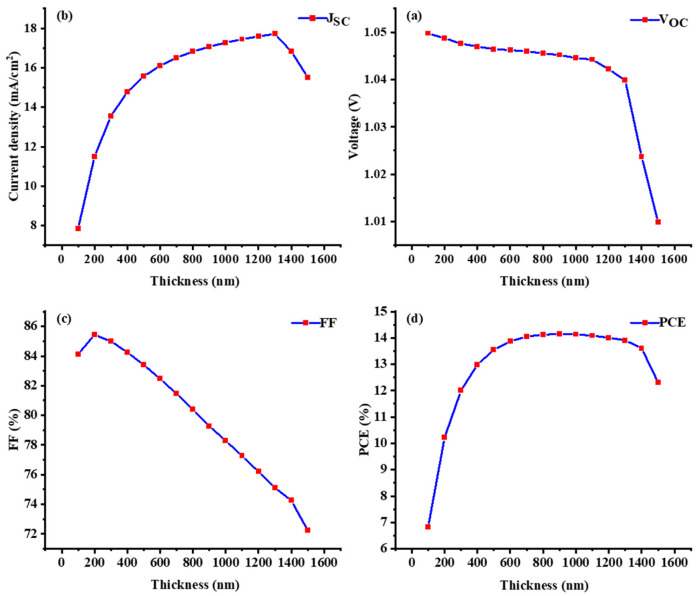
Variation in photovoltaic parameters with MAGeI_3_ thickness: (**a**) V_OC_, (**b**) J_SC_, (**c**) FF, and (**d**) PCE.

To evaluate the role of absorber thickness, the device performance was analyzed under different MAGeI_3_ thicknesses, as shown in Figure 7. When the absorber thickness varies from 100 to 1500 nm, the V_OC_ shows only a slight decrease from 1.05 to 1.01 V, whereas the J_SC_ changes much more obviously and reaches 17.80 mA/cm^2^ at 1300 nm. This trend is mainly attributed to the stronger light-harvesting capability of a thicker perovskite layer, which contributes to an increase in J_SC_. However, once the absorber layer becomes excessively thick, the transport path of carriers becomes longer, which increases the probability of recombination and causes J_SC_ to decline beyond 1300 nm. As shown in Figure 7c, the FF gradually decreases over the absorber thickness range of 200 to 1500 nm, which can be attributed to the longer carrier transport path and enhanced recombination loss in the thicker absorber layer within the SCAPS model. Finally, according to Figure 7d, the optimum efficiency of 14.29% is obtained when the absorber thickness is 900 nm.

### 3.4. Impact of the Doping Density of ETL and HTL

**Figure 8:**
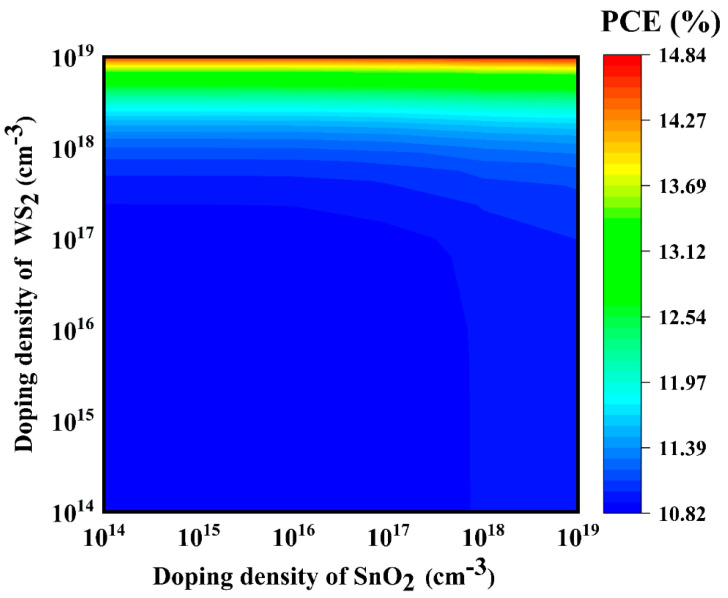
The efficiency of PSCs with different doping densities of WS_2_ and SnO_2_.

For device optimization, controlling the doping density in ETL (SnO_2_) and HTL (WS_2_) is essential. In our work, their effects are examined over the range of 1 × 10^14^ to 1 × 10^19^ cm^−3^, as presented in Figure 8. At relatively low doping levels, the device PCE increases as the density rises. At a doping density of 1 × 10^19^ cm^−3^ for SnO_2_ and WS_2_, the PCE reaches a maximum efficiency of 14.83%. Fundamentally, as the doping densities of SnO_2_ and WS_2_ increase, the built-in electric field near the MAGeI_3_ interfaces is strengthened, which facilitates electron–hole pair separation and improves device performance. Furthermore, the results indicate that, compared to the doping concentration of SnO_2_, an increase in the doping concentration of WS_2_ has a more significant impact on device performance.

### 3.5. Impact of MAGeI_3_ Absorber Defect Density

**Figure 9:**
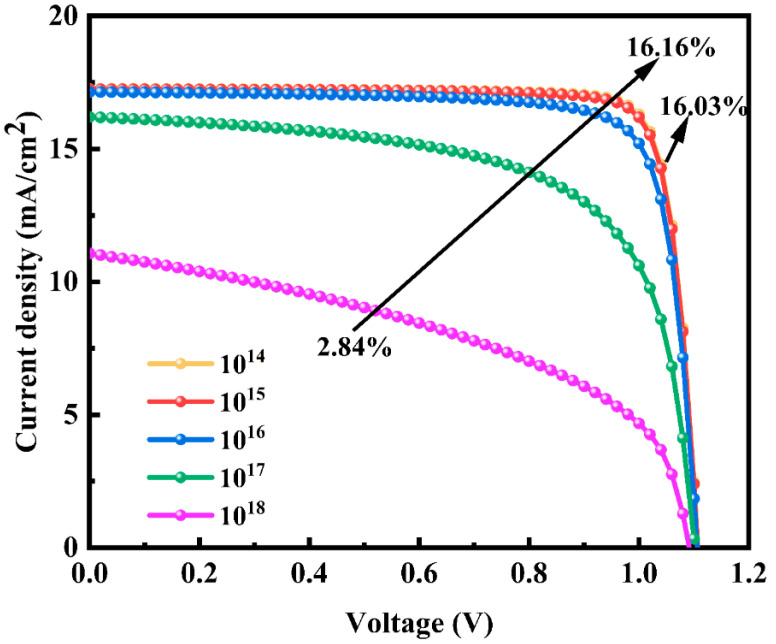
J-V curves with different MAGeI_3_ defect densities.

To evaluate the impact of bulk defects on device performance, the defect density in the MAGeI_3_ absorber is varied from 1 × 10^14^ to 1 × 10^18^ cm^−3^. As shown in Figure 9, increasing the defect density beyond 10^15^ cm^−3^ causes a significant deterioration in photovoltaic performance, and the PCE drops from 16.03% to 2.84% at 1 × 10^18^ cm^−3^. By comparison, reducing the defect density below 10^15^ cm^−3^ leads to only a slight improvement. This trend is attributed to the increased trap-assisted Shockley–Read–Hall recombination in the MAGeI_3_ absorber layer, since a higher defect density introduces more recombination-active trap states. Therefore, 1 × 10^15^ cm^−3^ is adopted as the optimized bulk defect density for the subsequent simulations.

### 3.6. Influence of Defect Densities at MAGeI_3_ Interfaces

**Figure 10:**
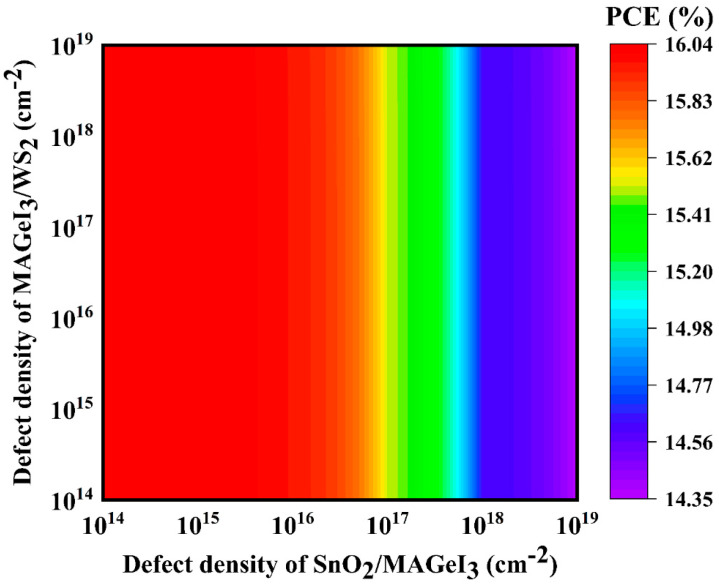
The efficiency of PSCs with different defect densities at the MAGeI_3_ interfaces.

Figure 10 shows that the defect densities at the SnO_2_/MAGeI_3_ and MAGeI_3_/WS_2_ interfaces have a significant effect on device efficiency. As the defect density decreases from 1 × 10^19^ to 1 × 10^15^ cm^−2^, the efficiency increases from 14.35% to 16.03%. However, when the defect density falls below 1 × 10^15^ cm^−2^, the improvement in efficiency becomes negligible. Moreover, the MAGeI_3_/WS_2_ interface has a stronger influence on device performance than the SnO_2_/MAGeI_3_ interface. This difference can be attributed to different carrier distributions, interfacial defect states, band alignment, and recombination processes at the two interfaces. At the MAGeI_3_/WS_2_ interface, interfacial defects can act as recombination centers for photogenerated carriers, which increases recombination loss and degrades device performance. Accordingly, the defect density of the MAGeI_3_ interfaces is set to 1 × 10^15^ cm^−2^ for the subsequent simulations.

### 3.7. Impact of ITO Work Function

**Figure 11:**
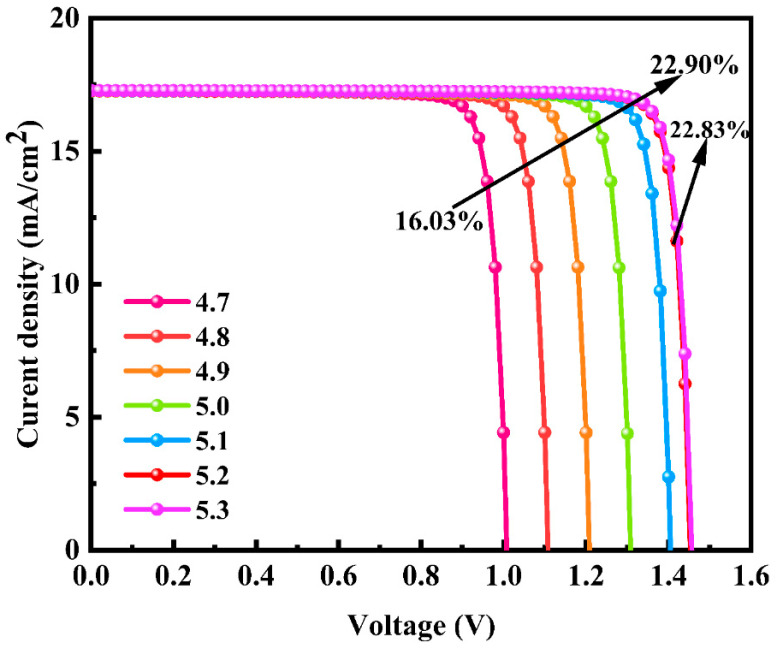
Comparison of J-V curves for PSCs with different ITO work functions.

In addition to transport layer parameters, the work function of ITO plays an important role in device performance. The work function of ITO can be effectively tuned through surface treatments, including plasma treatment, surface molecular modification, and chlorination [54,55,56]. As shown in Figure 11, increasing the ITO work function from 4.8 to 5.2 eV enhances the efficiency from 16.03% to 22.9%. Once the ITO work function exceeds 5.2 eV, its influence on device performance becomes negligible. When the work function falls below 5.2 eV, a pronounced energy level mismatch emerges at the ITO/WS_2_ interface, which induces a Schottky barrier and suppresses hole extraction. Therefore, the ITO work function should be tuned to 5.2 eV or larger, and the device can reach the optimum efficiency of 22.9%.

## 4. Conclusions

This research presents a simulation study of inverted MAGeI_3_ solar cells with a configuration of glass substrate/ITO/HTL/MAGeI_3_/ETL/Ag using SCAPS. Three HTLs and five ETLs have been carefully investigated. Under the simulation conditions investigated, WS_2_ provides the highest simulated performance among the considered HTLs, which is related to its favorable VBO with MAGeI_3_ and electron blocking ability. SnO_2_ provides the highest simulated performance among the considered ETLs, mainly because the band alignment at the SnO_2_/MAGeI_3_ interface helps suppress interfacial recombination and improve carrier selectivity. The effects of MAGeI_3_ absorber thickness, ETL and HTL doping densities, absorber defect density, interfacial defect density, and ITO work function were then systematically studied. The results indicate that the MAGeI_3_ layer achieves its optimum thickness at 900 nm. The optimal doping density of both WS_2_ and SnO_2_ is 1 × 10^19^ cm^−3^. It is important to maintain a low defect density of 1 × 10^15^ cm^−3^ in the absorber layer, and the defect densities at the MAGeI_3_ interfaces should be kept as 1 × 10^15^ cm^−2^ or lower. When the ITO work function is maintained at 5.2 eV, Schottky barrier formation at the ITO/WS_2_ interface is suppressed, allowing the device to achieve a peak efficiency of 22.9%.

Beyond these parameter optimizations, the simulation results provide design guidance for inverted MAGeI_3_ PSCs. The device performance is governed by the coupled effects of transport layer band alignment, absorber quality, interface recombination, and electrode contact properties. Therefore, further improvement requires simultaneous optimization of carrier-selective contacts, MAGeI_3_ film quality, and interface passivation. Compared with the previously reported experimental MAGeI_3_ solar cell with a PCE of 0.20%, the simulated efficiency obtained in this work suggests that inverted MAGeI_3_ PSCs still have considerable potential for performance improvement. It should be noted that the PCE of 22.9% was obtained from SCAPS simulation under the adopted material parameters and investigated parameter ranges. Therefore, this value should be regarded as the theoretical potential of the proposed device structure rather than a direct prediction of experimentally achievable efficiency. Further experimental studies are still needed to examine the practical feasibility of the proposed device structure and optimize the MAGeI_3_ film quality, interface quality, and relevant device parameters.

## Figures and Tables

**Table 1:** MAGeI_3_-based PSCs from recent theoretical studies [28,29,30,31].

Ref.	Device Structure	J_SC_ (mA/cm^2^)	V_OC_ (V)	FF (%)	PCE (%)
[28]	Au/Spiro-OMeTAD/MAGeI_3_/ZnOS/FTO	14.51	1.77	84.05	21.62
[29]	Au/Cu_2_O/MAGeI_3_/TiO_2_/FTO	15.55	1.34	82.73	18.01
[30]	Ag/Spiro-OMeTAD/MAGeI_3_/TiO_2_/ITO	11.23	1.18	89.51	11.88
[31]	Au/Cu_2_O/MAGeI_3_/ZnSe/FTO	14.22	1.37	89.99	17.61

**Table 2:** Initial parameters of all used materials [44,45,46,47].

Parameters	PCBM	MAGeI_3_	MoS_2_	WS_2_	PEDOT:PSS
Thickness, d (nm)	50	400	5	5	20
Band gap, E_g_ (eV)	2.4	1.9	1.55	1.85	1.6
Electron affinity, χ (eV)	4.0	3.98	3.8	3.55	3.4
Relative permittivity, ε	3.9	10.0	4.0	8.27	10.0
Effective conduction band density, N_C_ (cm^−3^)	1 × 10^21^	1 × 10^16^	7.5 × 10^17^	1.0 × 10^19^	1.0 × 10^21^
Effective valence band density, N_V_ (cm^−3^)	2 × 10^20^	1 × 10^15^	1.8 × 10^18^	1.4 × 10^19^	1.0 × 10^21^
Mobility of electron, μ_n_ (cm^2^/Vs)	0.2	16.2	100	60	1
Mobility of hole, μ_p_ (cm^2^/Vs)	0.2	10.1	150	95	40
Donor density, N_D_ (cm^−3^)	2.93 × 10^17^	1.0 × 10^9^	0	0	0
Acceptor density, N_A_ (cm^−3^)	0	1 × 10^9^	1 × 10^15^	1 × 10^16^	1 × 10^16^

**Table 3:** Interfacial defect parameters (neutral) [44].

Parameters	ETL/MAGeI_3_	MAGeI_3_/HTL
Defect density, N_t_ (cm^−2^)	1 × 10^15^	1 × 10^15^
Energy level (eV)	0.6	0.6

**Table 4:** Initial material parameters of ETLs [44].

Parameters	SnO_2_	TiO_2_	ZnO	IGZO
Thickness (nm)	30	30	30	30
E_g_ (eV)	3.50	3.20	3.20	3.05
χ (eV)	3.90	4.00	4.26	4.16
ε	9.0	9.0	9.0	10.0
N_C_ (cm^−3^)	2.2 × 10^17^	2.0 × 10^18^	2.0 × 10^18^	5.0 × 10^18^
N_V_ (cm^−3^)	2.2 × 10^16^	1.8 × 10^19^	1.8 × 10^19^	5.0 × 10^18^
μ_n_ (cm^2^/Vs)	20	20	200	15
μ_p_ (cm^2^/Vs)	10	10	5	0.2
N_D_ (cm^−3^)	1.0 × 10^17^	1.0 × 10^18^	1.5 × 10^17^	1.5 × 10^17^

**Table 5:** Comparison of simulated device performance parameters for different HTLs.

HTL	V_OC_ (V)	J_SC_ (mA/cm^2^)	FF (%)	PCE (%)
PEDOT:PSS	0.81	13.44	80.46	8.76
MoS_2_	0.88	13.27	80.58	9.41
WS_2_	0.89	13.28	80.04	9.46

**Table 6:** Comparison of simulated device performance parameters for different ETLs.

ETL	V_OC_ (V)	J_SC_ (mA/cm^2^)	FF (%)	PCE (%)
TiO_2_	1.04	14.55	62.72	9.49
SnO_2_	1.06	14.60	61.60	9.53
ZnO	0.92	14.40	57.90	7.68
PCBM	1.03	14.55	63.13	9.46
IGZO	0.98	14.45	62.05	8.78

## Data Availability

The original contributions presented in this study are included in the article. Further inquiries can be directed to the corresponding author.
